# Prevalence of Allergic Disorders among Primary School-Aged Children in Madinah, Saudi Arabia: Two-Stage Cross-Sectional Survey

**DOI:** 10.1371/journal.pone.0036848

**Published:** 2012-05-17

**Authors:** Mahmoud Nahhas, Raj Bhopal, Chantelle Anandan, Rob Elton, Aziz Sheikh

**Affiliations:** 1 Allergy and Respiratory Research Group, Centre for Population Health Sciences, The University of Edinburgh, Edinburgh, United Kingdom; 2 Edinburgh Ethnicity Health and Research Group, Centre for Population Health Sciences, Medical School, The University of Edinburgh, Edinburgh, United Kingdom; Johns Hopkins Bloomberg School of Public Health, United States of America

## Abstract

**Background:**

There are limited data on the epidemiology of allergic disorders in Saudi Arabia. Such data are needed for, amongst other things, helping to plan service provision at a time when there is considerable investment taking place in national healthcare development. We sought to estimate the prevalence of atopic eczema, allergic rhinitis and asthma in primary school children in Madinah, Saudi Arabia.

**Methods and Findings:**

We conducted a two-stage cross-sectional survey of schoolchildren in Madinah. Children were recruited from 38 randomly selected schools. Questionnaires were sent to the parents of all 6,139 6–8 year old children in these schools. These parental-completed questionnaires incorporated questions from the International Study of Asthma and Allergies in Childhood (ISAAC), which had previously been validated for use in Arab populations. We undertook descriptive analyses, using the Generalized Estimating Equation (GEE) to calculate 95% confidence intervals. The overall response rate was 85.9% (n = 5,188), 84.6% for girls and 86.2% for boys, respectively. Overall, parents reported symptoms suggestive of a history of eczema in 10.3% (95%CI 9.4, 11.4), rhinitis in 24.2% (95%CI 22.3, 26.2) and asthma in 23.6% (95%CI 21.3, 26.0) of children. Overall, 41.7% (95%CI 39.1, 44.4) of children had symptoms suggestive of at least one allergic disorder, with a substantial minority manifesting symptoms indicative of co-morbid allergic disease. Comparison of these symptom-based prevalence estimates with reports of clinician-diagnosed disease suggested that the majority of children with eczema and asthma had been diagnosed, but only a minority (17.4%) of children had been diagnosed with rhinitis. International comparisons indicated that children in Madinah have amongst the highest prevalence of allergic problems in the world.

**Conclusions:**

Symptoms indicative of allergic disease are very common in primary school-aged children in Madinah, Saudi Arabia, with figures comparable to the highest risk regions in the world.

## Introduction

The incidence and prevalence of allergic disorders has increased considerably in recent decades, so much so that allergic disorders are now amongst the most common chronic disorders of childhood [1]. Whilst major international epidemiological studies such as the International Study of Asthma and Allergy in Childhood (ISAAC) [2]–[3] and the European Community Respiratory Health Survey (ECRHS) [4] have greatly increased our understanding of allergic disease prevalence in many parts of the world, there remains a dearth of epidemiological data in relation to much of the Middle East, including Saudi Arabia. [5]. This is problematic, because it hampers understanding of these conditions in Saudi children, making planning of appropriate health provision difficult.

The limited available data in Saudi Arabia on allergic disorders are largely confined to asthma, with studies indicating that the prevalence in Saudi Arabia varies anywhere between 8–15% in children [6]–[7]. These studies are, however, relatively small-scale, are affected by methodological limitations such as the failure to make due adjustments for clustered sampling when estimating confidence intervals (CI), and are furthermore now somewhat dated.

We report on a large regional survey, which aimed to determine the prevalence of asthma and other allergic diseases among Saudi children using the validated Arabic version of the ISAAC questionnaire [8].

## Methods

### Ethics Statement

There is no health ethics committee for school-based research in the Madinah region. Permission to undertake this research was however obtained from the education authorities in Madinah (General Directors) and school health departments in the Ministry of Education, Riyadh and from the respective school head teachers. Parents were asked to give their signed informed consent. All data had identifiers removed to prevent the risk of a breach of confidentiality.

### Design

We conducted a two stage cross-sectional survey of the parents of primary school students aged 6–8 years in Madinah, which is one of the largest provinces in Saudi Arabia, in April 2009. The two stages involved sampling and recruiting schools and then children from within these schools. The methods were largely based on those used in the ISAAC studies (see: http://isaac.auckland.ac.nz/index.html for further details) and piloted by the research team in Madinah, Saudi Arabia in 2008.

### Setting

Madinah is a city in the west of Saudi Arabia It has an area of 589 Km^2^ and is about 190 Km from the Red Sea coast. It has a population of ∼1.5 million people from a total Saudi population of 21.4 million [9].

### Recruitment of Schools

A list of all government and private primary schools in Madinah was obtained from the General Directorate of Education in the Madinah region (i.e. both boys’ and girls’ departments respectively); these primary schools were responsible for providing education for children aged 6–12 years. Schools were then stratified (according to the geographical area and sex) and a random sample of 38 schools (9 schools for girls and 29 for boys) was approached. We invited the schools to participate with a letter of explanation to the head teachers outlining the purpose of the study and the procedures that were to be employed.

### Recruitment of Children

There were 33,270 male students (59.7%) and 22,412 female students (40.3%) aged 6–8years old in primary schools in Madinah (i.e. a total of 55,682 students). All students aged 6–8 years who were long-term residents in Madinah (i.e. for at least two years) and enrolled in the selected schools were eligible to participate. The parents of these children were sent, via the school, a letter explaining the rationale for the study and what it entailed, a consent form and a questionnaire, which they were invited to complete and return this to the class teacher. There were no reminders issued to non-responders.

### Questionnaire

Permission was obtained from Oman University to use the Arabic version of the validated ISAAC questionnaire [8]. The questionnaire was in two parts. Part one was the Arabic translated version of the core ISAAC questionnaire, which comprised 21 questions posed to parents relating to the prevalence of eczema, rhinitis and asthma in their children; the information requested included: 1) parental reports of symptoms of allergic diseases; 2) parental reports of diagnosed allergic diseases; and 3) parental reports of current symptoms of allergic disorders. The second part of the questionnaire contained questions relevant to possible environmental risk factors for the development of these conditions. These environmental questions were modified slightly to ensure that all questions were relevant to the Saudi context, for example, relating to housing conditions, exposure to animals and presence of animals at home, number of siblings and other people living in the house, and parental smoking. Data on these environmental risk factors will be reported separately in due course.

### Data Analysis

Data were coded, checked, entered into an Excel spreadsheet (version 2003) and then analysed using SPSS (Statistical Package for Social Sciences) (Version 16). Descriptive analysis was undertaken, expressing categorical data as numbers and percentages; to account for the two-stage sampling, Generalised Estimating Equations (GEE) were used to fit random effects logistic models to estimate and calculate 95% CI for prevalence; continuous data were summarised using means and standard deviations [10]. We report separate prevalence estimates for both males and females; we also calculated overall estimates in children to allow comparison with international data.

## Results

### Recruitment of Schools and Charactertistics

All 38 schools approached agreed to participate. In keeping with the wider preponderance of government-funded schools, 36/38 (94.7%) of these schools were government funded.

### Parental Response Rate

A total of 6,139 questionnaires were distributed for parental completion, of which 5,188 (85.9%) completed questionnaires were returned to the school and collected from the schools by the research team. Replies were received from the parents of 3585/4159 (86.2%) boys and 1603/1894 (84.6%) girls, respectively. Missing values for any question did not exceed 10.0% for any question.

### Characteristics of Respondents

The percentages of children in the age ranges 6–7, 7–8 and 8–9 years were: 16%, 36% and 48% for boys; and 17%, 30% and 52% for girls. The majority of the study population were Saudi nationals (80%); Table 1 summarises data on the characteristics of the families of participating children.

**Table 1:** Characteristics of the study population.

Variable	Males (n = 3585)	Females (n = 1603)	Total (n = 5188)
**Father’s age, years: mean (SD)**	43.6 (8.6)	44.4 (8.4)	43.8 (8.6)
**Mother’s age, years: mean (SD)**	35.2 (6.2)	36.2 (6.0)	35.5 (6.1)
**Father’s education (highest qualification) n (%)**			
None	260 (7.3%)	153 (9.5%)	413 (7.9%)
General education	1966 (54.8%)	922 (57.5%)	2888 (55.7%)
Higher education	1200 (33.5%)	459 (28.6%)	1659 (31.9%)
Missing	159 (4.4%)	69 (4.3%)	228 (4.4%)
**Mother’s education (highest qualification) n (%)**			
None	381 (10.6%)	208 (12.9%)	589 (11.4%)
General education	2035 (56.8%)	963 (60.1%)	2998 (57.8%)
Higher education	1057 (29.5%)	364 (22.7%)	1421 (27.4%)
Missing	112 (3.12%)	68 (4.2%)	180 (3.5%)
**Exposure to farm animals during pregnancy with this child? n (%)**			
Yes	155 (4.5%)	63 (3.9%)	218 (4.2%)
No	3266 (95.5%)	1467 (91.5%)	4733 (91.2%)
Missing	164 (4.6%)	73 (4.6%)	237 (4.6%)
**Was the child born in Madinah? n (%)**			
Yes	2999 (83.7%)	1318 (82.2%)	4317 (83.2%)
No	521 (14.5%)	257 (16.0%)	778 (15.0%)
Missing	65 (1.8%)	28 (1.7%)	93 (1.8%)
**Child’s birth weight in kg: mean (SD)**	2.94 (0.64)	2.91 (0.66)	2.93 (0.64)
**Did the mother breast feed? n (%)**			
Yes	2935 (81.9%)	1319 (82.3%)	4254 (82.0%)
No	567 (15.8%)	240 (15.0%)	807 (15.6%)
Missing	83 (2.3%)	44 (2.7%)	127 (2.4%)
**Birth order n (%)**			
First child	626 (17.5%)	226 (14.1%)	852 (16.4%)
Second child	587 (16.4%)	292 (18.2%)	879 (17.0%)
Third or greater	2296 (64.0%)	1034 (64.5%)	3330 (64.2%)
Missing	76 (2.1%)	51 (3.2%)	127 (2.4%)
**How many years has the child lived in Madinah? n (%)**			
More than one year	3354 (93.6%)	1487 (92.8%)	4841 (93.3%)
One year or less	47 (1.3%)	19 (1.2%)	66 (1.3%)
Missing	184 (5.1%)	97 (6.1%)	281 (5.4%)
**Does the father smoke? n (%)**			
Yes	828 (23.1%)	405 (25.3%)	1233 (23.8%)
No	2695 (75.2%)	1164 (72.6%)	3859 (74.4%)
Missing	62 (1.7%)	34 (2.1%)	96 (1.9%)
**Does the mother smoke? n (%)**			
Yes	30 (0.8%)	13 (0.8%)	43 (0.8%)
No	3483 (97.2%)	1562 (97.4%)	5045 (97.2%)
Missing	72 (2.0%)	28 (1.8%)	100 (2.0%)
**Did the mother smoke in the child’s 1st year of the life? n (%)**			
Yes	24 (0.6%)	14 (0.9%)	38 (0.7%)
No	3491 (97.4%)	1552 (96.8%)	5043 (97.2%)
Missing	70 (2.0%)	37 (2.3%)	107 (2.1%)
**Number of smokers in the household n (%)**			
No smokers	2675 (74.6%)	1156 (72.1%)	3831 (73.8%)
one or more smokers	838 (23.4%)	410 (25.6%)	1248 (24.1%)
Missing	72 (2.0%)	37 (2.3%)	109 (2.1%)
**Was there a cat at home in the child’s 1^st^ year of life? n (%)**			
Yes	160 (4.5%)	67 (4.2%)	227 (4.4%)
No	3345 (93.3%)	1496 (93.3%)	4841 (93.3%)
Missing	80(2.2%)	40(2.5%)	120 (2.3%)
**Was there a cat at home in the last 12 months? n (%)**			
Yes	224 (6.3%)	91 (5.7%)	315 (6.1%)
No	3292 (91.8%)	1470 (91.7%)	4762 (91.8%)
Missing	69 (1.9%)	42 (2.6%)	111 (2.2%)
**Did the child have antibiotics in the 1st year? n (%)**			
Yes	2002 (55.8%)	926 (57.8%)	2928 (56.4%)
No	1384 (38.6%)	597 (37.2%)	1981 (38.2%)
Missing	199 (5.6%)	80 (5.0%)	279 (5.4%)
**Did the child have paracetamol during the first year of life? n (%)**			
Yes	3011 (84%)	1232 (77.0%)	4243 (82.0%)
No	441 (12.3%)	305 (19.0%)	746 (14.4%)
Missing	133 (3.7%)	66 (4.1%)	199 (3.8%)
**On average, how many times you have given your child paracetamol in last 12 months? n (%)**			
Once a week	634 (17.7%)	373 (23.3%)	1007 (19.4%)
Once a month	1765 (49.2%)	740 (46.2%)	2505 (48.3%)
Once a year	860 (24.0%)	258 (16.1%)	1118 (21.5%)
Missing	326 (9.1%)	232 (14.5%)	558 (11.0%)
**How many hours a day does your child watch TV? n (%)**			
<3 hours	2167 (60.5%)	960 (60.0%)	3127 (60.3%)
>3 hours	1230 (34.3%)	557 (34.7%)	1787 (34.4%)
Missing	188 (5.2%)	86 (5.4%)	274 (5.3%)
**How many times a week did your child take exercise? n (%)**			
Never	2262 (63.1%)	1253 (78.2%)	3515 (67.8%)
Once or twice a week	774 (21.6%)	191 (12.0%)	965 (18.6%)
Three times or more	298 (8.3%)	39 (2.4%)	337 (6.5%)
Missing	251 (7.0%)	120 (7.5%)	371 (7.2%)
**Does your home have air conditioning? n (%)**			
Electric fan only	28 (0.8%)	24 (1.5%)	52 (1.0%)
Water system only	219 (6.1%)	516 (32.2%)	735 (14.2%)
Freon system only	3106 (86.6%)	976 (61.0%)	4082 (78.7%)
Both Freon & water systems	55 (1.5%)	19 (1.2%)	74 (1.4%)
Missing	177 (5.0%)	68 (4.2%)	245 (4.7%)
**How many times on average does a truck pass the street adjacent to your home? n (%)**			
Rarely	2350 (65.6%)	1105 (69.0%)	3455 (66.6%)
Frequently	1087 (30.3%)	433 (27.0%)	1520 (29.3%)
Missing	148 (4.1%)	65 (4.1%)	213 (4.1%)
**What is the fuel normally used in cooking in your household? n (%)**			
Electricity only	154 (4.3%)	49 (3.1%)	203 (4.0%)
Gas only	3270 (91.2%)	1484 (92.6%)	4754 (91.6%)
Wood fire or coal	3 (0.1%)	7 (0.4%)	10 (0.2%)
Both Electricity & gas	48 (1.3%)	20 (1.2%)	68 (1.3%)
Missing	110 (3.1%)	43 (2.7%)	153 (2.9%)
**Diet – how many times a week does your child eat the following?**			
**Meat n (%)**			
Never	244 (7.0%)	135 (8.4%)	379 (7.3%)
Once or twice a week	1169 (32.6%)	553 (34.5%)	1722 (33.4%)
Three or more a week	2052 (57.2%)	859 (53.6%)	2911 (56.1%)
Missing	120 (3.3%)	56 (3.5%)	176 (3.4%)
**Fish** **n (%)**			
Never	1631 (45.5%)	744 (46.4%)	2375 (45.8%)
Once or twice a week	1606 (44.8%)	689 (43.0%)	2295 (44.2%)
Three or more a week	171 (4.8%)	76 (4.7%)	247 (4.8%)
Missing	177 (4.9%)	94 (6.0%)	271 (5.2%)
**Fruit n (%)**			
Never	534 (14.9%)	173 (10.8%)	707 (13.6%)
Once or twice a week	1500 (41.8%)	686 (42.8%)	2186 (42.2%)
Three or more a week	1431 (40.0%)	677 (42.2%)	2108 (40.6%)
Missing	120 (3.3%)	67 (4.2%)	187 (3.6%)
**Vegetables** **n (%)**			
Never	444 (12.4%)	152 (9.5%)	596 (11.5%)
Once or twice a week	1313 (36.6%)	580 (36.2%)	1893 (36.5%)
Three or more a week	1623 (45.3%)	779 (48.6%)	2402 (46.3%)
Missing	205 (5.7%)	92 (5.7%)	297 (5.7%)
**Legumes** **n (%)**			
Never	1180 (33.0%)	483 (30.1%)	1663 (32.1%)
Once or twice a week	1555 (43.4%)	680 (42.4%)	2235 (43.1%)
Three or more a week	673 (18.8%)	347 (21.7%)	1020 (19.7%)
Missing	177 (4.9%)	93 (5.8%)	270 (5.2%)
**Cereal n (%)**			
Never	97 (2.7%)	41 (2.6%)	138 (2.7%)
Once or twice a week	394 (11.0%)	181 (11.3%)	575 (11.1%)
Three or more a week	2935 (82.0%)	1291 (80.5%)	4226 (81.5%)
Missing	159 (4.4%)	90 (5.6%)	249 (4.8%)
**Pasta n (%)**			
Never	764 (21.3%)	338 (21.1%)	1102 (21.3%)
Once or twice a week	1915 (53.4%)	862 (53.8%)	2777 (53.5%)
Three or more a week	755 (21.1%)	335 (21.0%)	1090 (21.0%)
Missing	151 (4.2%)	68 (4.2%)	219 (4.2%)
**Rice** **n (%)**			
Never	136 (3.8%)	50 (3.1%)	186 (3.6%)
Once or twice a week	744 (20.8%)	336 (21.0%)	1080 (20.8%)
Three or more a week	2549 (71.1%)	1133 (70.7%)	3682 (71.0%)
Missing	156 (4.4%)	84 (5.2%)	240 (4.6%)
**Butter n (%)**			
Never	2072 (57.8%)	943 (58.8%)	3015 (58.1%)
Once or twice a week	942 (26.3%)	373 (23.3%)	1315 (25.3%)
Three or more a week	343 (9.6%)	168 (10.5%)	511 (10.0%)
Missing	228 (6.4%)	119 (7.4%)	347 (6.7%)
**Margarine** **n (%)**			
Never	2225 (62.1%)	1008 (62.9%)	3233 (62.3%)
Once or twice a week	657 (18.3%)	278 (17.3%)	935 (18.0%)
Three or more a week	378 (10.5%)	158 (9.9%)	536 (10.3%)
Missing	325 (9.1%)	159 (10.0%)	484 (9.4%)
**Nuts** **n (%)**			
Never	1905 (53.1%)	806 (50.3%)	2711 (52.3%)
Once or twice a week	1168 (32.6%)	550 (34.3%)	1718 (33.1%)
Three or more a week	295 (8.2%)	148 (9.2%)	443 (8.5%)
Missing	217 (6.1%)	99 (6.2%)	316 (6.1%)
**Potatoes** **n (%)**			
Never	284 (8.0%)	144 (9.0%)	428 (8.3%)
Once or twice a week	1662 (46.4%)	690 (43.0%)	2352 (45.3%)
Three or more a week	1488 (41.5%)	703 (43.9%)	2191 (42.2%)
Missing	151 (4.2%)	66 (4.1%)	217 (4.2%)
**Milk** **n (%)**			
Never	300 (8.4%)	127 (8.0%)	427 (8.3%)
Once or twice a week	773 (21.6%)	358 (22.3%)	1131 (21.8%)
Three or more a week	2383 (66.5%)	1063 (66.3%)	3446 (66.4%)
Missing	129 (3.6%)	55 (3.4%)	184 (3.5%)
**Eggs n (%)**			
Never	391 (11.0%)	186 (11.6%)	577 (11.1%)
Once or twice a week	1353 (37.7%)	587 (36.6%)	1940 (37.4%)
Three or more a week	1697 (47.3%)	751 (46.8%)	2448 (47.2%)
Missing	144 (4.0%)	79 (5.0%)	223 (4.3%)
**Fast food n (%)**			
Never	2088 (58.3%)	1020 (63.6%)	3108 (60.0%)
Once or twice a week	1033 (28.8%)	377 (23.5%)	1410 (27.2%)
Three or more a week	281 (7.8%)	119 (7.4%)	400 (7.7%)
Missing	183 (5.1%)	87 (5.5%)	270 (5.2%)

### Overall Prevalence of Symptoms Suggestive of Allergic Problems

Overall, 2,163 children (41.7%, 95%CI 39.1, 44.4) had symptoms suggestive of at least one allergic problem.

### Prevalence of Parental Reports of Symptoms Suggestive of, Current and Clinician-diagnosed Eczema, Rhinitis and Asthma

In the section below we summarise data on the overall prevalence of parental reports of symptoms suggestive of, diagnosed and current eczema, rhinitis and asthma. The 95%CI for these estimates are presented in Table 2 together with sex-related variations and corresponding 95%CIs.

**Table 2:** Prevalence of parental reports of symptoms of, diagnosed and current allergic disease in 6–8 year old children.

Parental-reported outcomes	Females n, percent (95%CI)	Males n, percent (95%CI)	Total n, percent (95%CI)
**Ever had symptoms of eczema**	148, 9.2% (7.5, 11.3)	389, 10.9% (9.8, 12.0)	537, 10.4% (9.4, 11.4)
**Ever had symptoms of rhinitis**	300, 18.7% (16.9, 20.7)	957, 26.7% (25.0, 28.5)	1257, 24.2% (22.3, 26.2)
**Ever had symptoms of asthma**	351, 21.9% (17.4, 27.1)	874, 24.4% (22.0, 26.9)	1225, 23.6% (21.3, 26.0)
**Diagnosed eczema**	186, 11.6% (9.8, 13.7)	540, 15.1% (12.9, 17.5)	726, 14.0% (12.2, 15.9)
**Diagnosed rhinitis**	52, 3.2% (2.4, 4.4)	164, 4.6% (4.0, 5.2)	216, 4.2% (3.6, 4.7)
**Diagnosed asthma**	196, 12.2% (10.1, 14.7)	607, 16.9% (15.5, 18.4)	803, 15.5% (14.1, 17.0)
**Eczema symptoms in last 12 months**	122, 7.6% (5.9, 9.8)	332, 9.3% (8.2, 10.5)	454, 8.8% (7.8, 9.8)
**Rhinitis symptoms in last 12 months**	229, 14.3% (12.2, 16.7)	716, 20.0% (18.3, 21.7)	945, 18.2% (16.6, 20.0)
**Asthma symptoms in last 12 months**	113, 7.0% (5.0, 9.9)	416, 11.6% (10.5, 12.9)	529, 10.2% (8.9, 11.7)

#### Eczema

Overall, 10.4% of parents reported a history of a chronic (≥6 months) itchy rash in their children. The prevalence of parental reports of symptoms of itchy rash at any time in the last 12 months was 8.8%. There were parental reports of clinician-diagnosed eczema in 14.0% of children.

#### Rhinitis

Symptoms suggestive of ever-having had allergic rhinitis were common, affecting 24.2% of children overall, with an estimated 18.2% experiencing symptoms of sneezing in the last 12 months. The prevalence of children with parental reports of ever having a clinician diagnosis of allergic rhinitis was 4.2% overall.

#### Asthma

The overall prevalence of ever-having had wheeze or whistling in the chest was 23.6%, with reports of symptoms of asthma in the previous 12 months in 10.2% of children. The overall prevalence of children with parental reports of ever having been diagnosed with asthma was 15.5%.

### Co-morbid Allergic Disease

**Figure 1:**
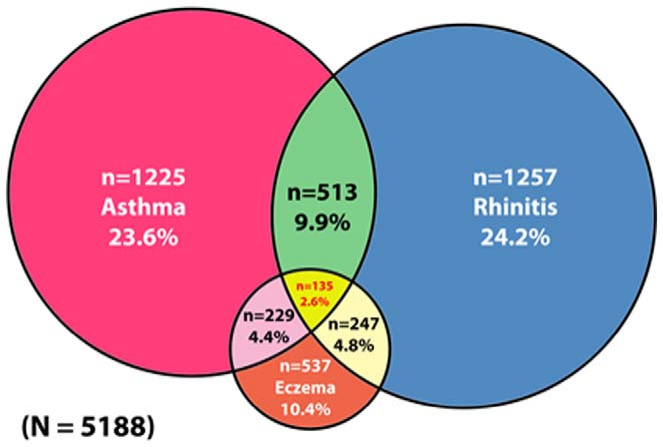
Venn diagram of patterns of co-morbid allergic disease in 6–8 year olds in Madinah, Saudi Arabia.

Whilst the majority (1447/2163; 66.9%) of children had symptoms suggestive of only one allergic condition, as demonstrated in Table 3 and Figure 1 a substantial minority of children had symptoms suggestive of co-morbid allergic disease.

**Table 3:** Prevalence of symptoms of co-morbid allergic disease in 6–8 year old children in Madinah, Saudi Arabia.

Co-morbidities	Females n, percent (95%CI)	Males n, percent (95%CI)	Total n, percent (95%CI)
Rhinitis and asthma	114, 7.1% (5.4, 9.3)	399, 11.1% (10.1, 12.2)	513, 9.9% (8.7, 11.1)
Asthma and eczema	64, 4.0% (3.1, 5.2)	165, 4.6% (3.9, 5.4)	229, 4.4% (3.8, 5.1)
Rhinitis and eczema	61, 3.8% (3.1, 4.7)	186, 5.2% (4.6, 5.9)	247, 4.8% (4.2, 5.4)
Eczema, rhinitis and asthma	30, 1.9% (1.3, 2.6)	105, 2.9% (2.5, 3.4)	135, 2.6% (2.2, 3.0)

### International Comparisons

**Figure 2:**
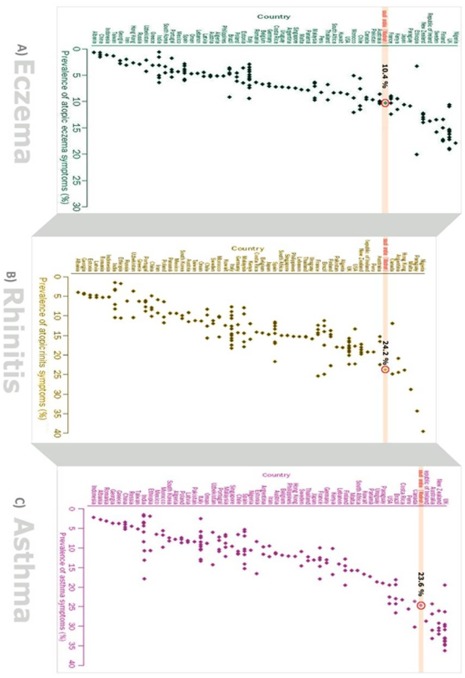
Comparison of prevalence of parental reports of symptoms of: a) eczema; b) rhinitis symptoms; and c) asthma in 6–8 year old Saudi children compared with 6–7 year old children in ISAAC participating countries in 1998 survey (2) (Saudi highlighted).

Figure 2 compares the prevalence estimates obtained from our study with data from other parts of the world, these revealing that Madinah is internationally a very high prevalence region.

## Discussion

This is, we believe, the largest study on the prevalence of allergic disease in school-aged children ever undertaken in the Arab world. The findings indicate that the prevalence of these problems is very high – affecting over 40% of children – within the first eight years of life. This is concerning given the considerable disease burden associated with these conditions, both to individuals and to society more generally [11]–[14].

### Strengths and Limitations of this Study

The main strengths of this study include the large sample size and the very high response rate, which is likely to be due to the combinaton of achieving good support from the participating schools and the fact that we were undertaking work in a population that has not previously been investigated, hence research fatigue was not an issue. Unlike the majority of previous studies, we did not confine ourselves to studying the prevalence of asthma [6]–[7]; [15]–[18]. Rather, we looked at other most common allergic problems, namely eczema and rhinitis; we also studied the co-morbidity between these conditions. The use of a validated instrument was an additional strength, particularly since this offered the opportunity to compare prevalence estimates for children from Madinah with children of a similar age from across the world (Figure 2) [19].

This major limitation with this work is that it was conducted in only one city in Saudi Arabia in children of one age group; these findings may therefore not be generalisable to other sections of Saudi society. It is important that similar studies are now conducted in other urban and rural locations in Saudi Arabia in children of other age groups. There is also the need to build on this work and monitor allergic disease trends in the Madinah region [20]. Finally, as we had no data that allowed us to compare the characteristics of responders and non-responders, care must be taken in seeking to extrapolate data from this work across the entire Madinah region.

### Comparison with the Wider Literature

The limited previous literature has suggested that the prevelance of asthma in Middle Eastern countries is lower than in “developed” countries[6]–[7]; [15]–[18]. a finding that is challenged by our study. Our study also proints to the high prevealcne of eczema and rhinitis in these children (Figure 2). This work has also highlighted the issue of possible under-diagnosis of rhinitis, and possibly also asthma, which echoes the findings from previous studies [21]–[22].

### Implications for Public Health Policy, Research and Practice

This work has identified the high prevalence of allergic disorders in Madinah and given the fact that these conditions are likely to continue to pose a significant burden to both individuals and society over the lifecourse, this suggests that allergic problems are likely to be an important public health consideration in Saudi Arabia for many years to come [13]–[14]; [23]. Given the very high prevalences of disease found in Madinah, it is important that this work is now extended to other Saudi regions; it is also important that attempts are made to understand key potentially modifiable environmental risk factors, which we will be reporting on in due course. It is furthermore also important that this population-based work is repeated in due course to allow disease trends to be determined [24]–[26]. Comparing the prevalence of symtomatic and clinician-diagnosed disease suggests that there may be substantial under-diagnosis of allergic rhinitis and to a lesser extent asthma. There is therefore a need for work to be now undertaken to verify this and, if confirmed, take steps to impove diagnostic capacity and accuracy, particualrly in community-based clinical seetings. Finally, there is the need for related work investigating the quality of care provided to children with allergic problems as this has been found to be wanting in other parts of the world [21]–[22].

### Conclusions

This large study of allergic disease prevalence in primary school-aged children in Madinah, Saudi Arabia has found that over 40% of children manifest symptoms indicative of allergic disease prevalence within the first eight years of life, these figures ranking amongst the highest in the world. More work is now needed on assessing the prevalence of allergic problems in other parts of Saudi Arabia, other age groups, and in monitoring disease trends. Given these very high figures, the Saudi Government needs to give careful consideration to developing health and educational policies that ensure the effective treatment of these children whilst minimising the impact of these conditions on educational performance; there is furthermore a need to understand better what is driving this epidemic in Saudi children and prioritise the search for effective primary prevention strategies.
